# The Impact of Volume Overload on the Longitudinal Change of Adipose and Lean Tissue Mass in Incident Chinese Peritoneal Dialysis Patients

**DOI:** 10.3390/nu14194076

**Published:** 2022-09-30

**Authors:** Jack Kit-Chung Ng, Gordon Chun-Kau Chan, Kevin Ka-Ho Kam, Na Tian, Win Hlaing Than, Phyllis Mei-Shan Cheng, Man-Ching Law, Wing-Fai Pang, Cheuk-Chun Szeto, Philip Kam-Tao Li

**Affiliations:** 1 Carol and Richard Yu Peritoneal Dialysis Research Centre, Department of Medicine & Therapeutics, Prince of Wales Hospital, The Chinese University of Hong Kong, Hong Kong 999077, China; 2 Division of Cardiology, Department of Medicine & Therapeutics, Prince of Wales Hospital, Hong Kong 999077, China; 3 Department of Nephrology, General Hospital of Ningxia Medical University, Yinchuan 750004, China; 4 Li Ka Shing Institute of Health Sciences (LiHS), Faculty of Medicine, The Chinese University of Hong Kong, Hong Kong 999077, China

**Keywords:** body composition, volume overload, muscle wasting, adipose tissue, bioimpedance, echocardiography, peritoneal dialysis

## Abstract

Patients treated with peritoneal dialysis (PD) experience complex body composition changes that are not adequately reflected by traditional anthropometric parameters. While lean and adipose tissue mass can be readily assessed by bioimpedance spectroscopy (BIS), there is concern about the potential confounding effect of volume overload on these measurements. This study aimed to assess the influence of fluid status (by echocardiography) on body composition parameters measured by BIS and to describe the longitudinal changes in adipose and lean tissue mass. We conducted a prospective observational study in a tertiary hospital. Incident Chinese PD patients underwent baseline echocardiography and repeated BIS measurements at baseline and 12 months later. Among 101 PD patients, lean tissue index (LTI) or fat tissue index (FTI) was not associated with echocardiographic parameters that reflected left ventricular filling pressure (surrogate of volume status). Sixty-eight patients with repeated BIS had a significant increase in body weight and FTI, while LTI remained similar. Gains in fat mass were significantly associated with muscle wasting (beta = −0.71, *p* < 0.0001). Moreover, progressive fluid accumulation independently predicted decrease in FTI (beta = −0.35, *p* < 0.0001) but not LTI. Body composition assessments by BIS were not affected by fluid status and should be considered as part of comprehensive nutrition assessment in PD patients.

## 1. Introduction

Peritoneal dialysis (PD) and hemodialysis (HD) represent two important modalities of renal replacement therapy for patients with end stage renal disease (ESRD). Hong Kong has adopted the ‘PD-first’ policy for 35 years because PD is a home-based and cost-effective therapy that provides continuous removal of uremic toxin and fluid [1]. When compared with in-center thrice-weekly HD, PD may provide comparable patient survival, better preserved residual renal function, and improved quality of life [2].

The latest guideline of International Society for Peritoneal Dialysis (ISPD) suggested that clinicians should not merely focus on achieving a specific target of urea clearance (classically represented by Kt/V) but also aim at optimizing volume and nutrition status in order to deliver high-quality PD [3]. Nutrition assessments in daily clinical practices typically rely on anthropometric measurements (e.g., body weight (BW) or body mass index (BMI)) and serum albumin, but these methods are not without limitations. First, an increase in BW does not help clinicians distinguish between an increase in adiposity and excessive fluid, both of which are common and frequently coexist in dialysis patients [4,5,6]. Second, although serum albumin is a well-recognized predictor of mortality in PD patients [7,8], its performance as nutritional index may be hampered given its close association with systemic inflammation and volume overload [9].

Changes in body composition and nutrition status may occur shortly after the initiation of dialysis. Choi et al. showed that incident PD patients gained weight significantly had increase in both visceral and subcutaneous fat as early as in the first six months of dialysis [10]. Patients with lower fat mass tended to gain more fat in due course [10]. An earlier analysis from the Netherlands Cooperative Study on the Adequacy of Dialysis (NECOSAD) study showed that by measuring skinfold thickness, incident PD patients were found to have significant increase in body fat compared to their HD counterparts, while lean body mass (LBM) remained similar over time in both groups [11]. On the other hand, volume overload (measured bioimpedance technique) was present in more than half of patients at the beginning of PD but tended to improve in the first year [12,13]. Even in the absence of clinical symptoms, volume overload was negatively correlated with serum albumin and significantly associated with mortality [13]. While weight gain was common after PD, such changes could be contributed by fluid accumulation together with subclinical muscle wasting [14].

Given the complex changes in lean tissue, adipose tissue and fluid in ESRD, it is not surprising that the ideal method to measure and monitor body composition remains controversial. Although dual-energy X-ray absorptiometry (DEXA) and isotope dilution scan are regarded as the gold standard, they are expensive and not easily accessible in routine practice. In the last decade, bioimpedance device has become popular in dialysis units because it allows simple and convenient assessment of body composition in an outpatient setting [15]. Specifically, bioimpedance enables non-invasive and highly reproducible quantification of fluid in different body compartments. Importantly, accumulating evidence has suggested that volume overload and muscle wasting measured by bioimpedance study were significantly associated with lower survival in PD patients [12,13,16,17]. However, the interaction between body compartments was seldom studied.

On the other hand, there is concern about the validity of bioimpedance in the assessment of LBM in overhydrated patients. A previous study reported a positive correlation between LBM measured by bioimpedance and left ventricular end-diastolic diameter (LVEDD) in a small cohort of PD patients [18]. Nevertheless, LVEDD was not a good surrogate for volume status because structural or valvular heart disease may cause ventricular dilation in the absence of hypervolemia [19,20]. Additionally, the bioimpedance technique (bioelectrical impedance analysis [BIA]) used in that study was not entirely equivalent to the more recent bioimpedance spectroscopy (BIS), which assumed a physiological three-compartment model consisting of normally hydrated lean tissue mass (LTM), normally hydrated adipose tissue mass (ATM) and volume of overhydration (OH) [21].

In the present study, we aimed to assess the influence of fluid status (by contemporary echocardiographic parameters) on body composition measurements by BIS in Chinese PD patients. In addition, we described the longitudinal changes in adipose and lean tissue mass and sought to identify potential predicting factors, in particular, hydration status.

## 2. Materials and Methods

### 2.1. Study Design

The present study was a secondary analysis of a prospective observational study, which primarily investigated the relationship between subclinical systolic dysfunction and volume overload [22]. We recruited consecutive incident PD patients from a tertiary hospital in Hong Kong from December 2018 to March 2021. Patients were excluded if they had pre-existing atrial fibrillation or valvular heart disease, or suffered from acute coronary syndrome or systemic infection that required hospitalization within 4 weeks before enrollment. Patients who had metallic prosthesis or pacemaker implantation were contraindicated for BIS and thus excluded. Included patients underwent baseline speckle-tracking echocardiography and BIS during PD training. Standard peritoneal equilibration test (PET) was performed 6–8 weeks later to determine peritoneal solute transfer rate (denoted by ratio of dialysate to plasma concentration of creatinine at the fourth hour (D/Pcr)). BIS were repeated 12 ± 6 months after enrollment. This study was approved by the Joint Chinese University Hong Kong-New Territories East Cluster Clinical Research Ethics Committee (CREC Ref. No.: 2018.361 and 2019.623). All study procedures adhered to the Declaration of Helsinki.

### 2.2. Data Collection

Baseline demographics including age, sex, causes of renal failure, and presence of diabetes were collected at the initiation of PD. Charlson Comorbidity Index (CCI) was calculated to represent the burden of comorbidities [23]. Peritoneal glucose exposure (grams per day) was defined as the product of the total dwell volume per day and the glucose concentration of each exchange. PD prescriptions were reviewed every 3 months and any change in dwell volume and PD fluid concentration were recorded. As a result, time-averaged peritoneal glucose exposure was calculated as the weighted mean of daily glucose exposure of each patient, with weights representing the time elapsed from the last measurement.

Blood samples were collected during the day of baseline and follow-up bioimpedance exam, respectively. Plasma concentration of hemoglobin, albumin, creatinine, fasting glucose and cholesterol, and C-reactive protein (CRP) were measured by local laboratory. In addition, plasma *N*-terminal pro-brain natriuretic peptide (NT-proBNP) was measured at baseline using enzyme-linked immunosorbent assay (ELISA) (Biomedica, Vienna, Austria) as a surrogate marker of volume status [24,25].

Dialysis adequacy was assessed by 24 h collection of peritoneal dialysate and urine. Weekly Kt/V and normalized protein nitrogen appearance (NPNA) were determined by standard method [26]. These parameters were first calculated 6–8 weeks after stabilization on PD (typically on the day of PET), then every 6 months until permanent change in renal replacement modality or death. Time-averaged values of these parameters were calculated by the same way mentioned above.

### 2.3. Echocardiography

At baseline, all subjects underwent transthoracic echocardiographic studies (TTE) by Philips IE33 system (Phillips Medical Systems, Andover, MA, USA). TTE images were acquired using the IE33 with an S5-1 transducer or EPIQ7 X5-1 transducer. All images were interpreted by a single echocardiography specialist who was blinded from clinical details.

Left ventricular ejection fraction (LVEF) was assessed by the modified Simpson’s biplane method. Other parameters such as left ventricular mass index (LVMI), left atrial volume index (LAVi), and diastolic function were determined according to the recommendations by the American Society of Echocardiography [27]. With the sample volume placed at medial and lateral mitral annulus in four-chamber apical view, pulsed-wave tissue Doppler imaging was used to measure the peak diastolic mitral inflow velocity (E.) and mitral annulus early diastolic tissue velocity (e’).

### 2.4. Body Composition Measurement

We used a validated multifrequency BIS (Body Composition Monitor, Fresenius Medical Care, Bad Homburg, Germany) to determine the body composition of subjects at baseline and one year later. This device applied small amplitude, alternating electrical current at 50 different frequencies (from 5 k to 1 M kHz) via two contact electrodes attached to patients in supine position. Body composition parameters (OH, LTM and fat tissue mass) were then automatically computed by mathematical algorithm based on the three-compartment model [21]. Excessive fluid can be expressed in absolute terms (OH, in liters), which was the difference between measured extracellular water (ECW) and predicted ECW assuming euvolemic tissue; or relative terms (relative hydration index (RHI), in percentage), in which OH was adjusted by ECW. Mild and severe volume overload was defined as RHI > 7 to 15% and RHI > 15%, respectively. Similarly, LTM and fat tissue mass were divided by square of height (in meters) and expressed as lean tissue index (LTI) and fat tissue mass (FTI). Patients were defined to have normal LTI or FTI if their LTI or FTI lied between 10th and 90th percentile of healthy Asian control [28]. BIS were performed with PD fluid in situ by the same research nurse with the same device to minimize inter-observer variability.

### 2.5. Statistical Analyses

Continuous variables were presented as mean ± standard deviation (SD) or median (inter-quartile range (IQR)). Continuous data were compared by independent t-test or Mann–Whitney U test depending on normality. Categorical variables were presented as percentages and compared by Chi-square test. The impact of hydration status on body composition measurements were evaluated by performing linear regression between echocardiographic parameters, NTproBNP and BIS parameters. The ratio of peak early mitral inflow velocity to mitral annulus early diastolic velocity (E/e’) and left atrial volume index (LAVi) were selected as the surrogate of LV filling pressure [19,20], while LVEF was the traditional marker of systolic function. Regression model involving echocardiographic parameters was adjusted for age, gender and body surface area (BSA) given their potential influence on these parameters [29]. Plasma NTproBNP was natural log-transformed due to skewness of data.

Changes in body composition parameters were explored by paired-t test or Wilcox signed rank test where appropriate. We then constructed separate linear regression models to identify the potential predictors of changes in LTI and FTI over 12 months, respectively. The first model consisted of parameters measured only at baseline, while the second model incorporated parameters measured at baseline and during follow-up. These parameters included demographic factors (age, gender, CCI), laboratory values (albumin, CRP, glucose, cholesterol), dialysis characteristics (PD modality, peritoneal glucose exposure, D/Pcr, dialysis adequacy and residual urine volume), and body composition measurements (BMI, LTI, FTI). They were first evaluated in univariable analyses. Variables which achieved a significance of *p* < 0.1, together with age and gender, were analyzed in a multivariable regression model using backward stepwise elimination. Multicollinearity was checked by estimation of variable inflation factor. In addition, we performed sensitivity analyses that excluded patients who required hospitalization during the study period.

All statistical analyses were performed by SPSS version 27.0 (IBM Corporation, Armonk, NY, USA) and Stata version 15 (StataCorp LP, College Station, TX, USA). A *p*-value of less than 0.05 was considered significant. All probabilities were two-tailed.

## 3. Results

### 3.1. Baseline Characteristics of Study Participants

**Figure 1:**
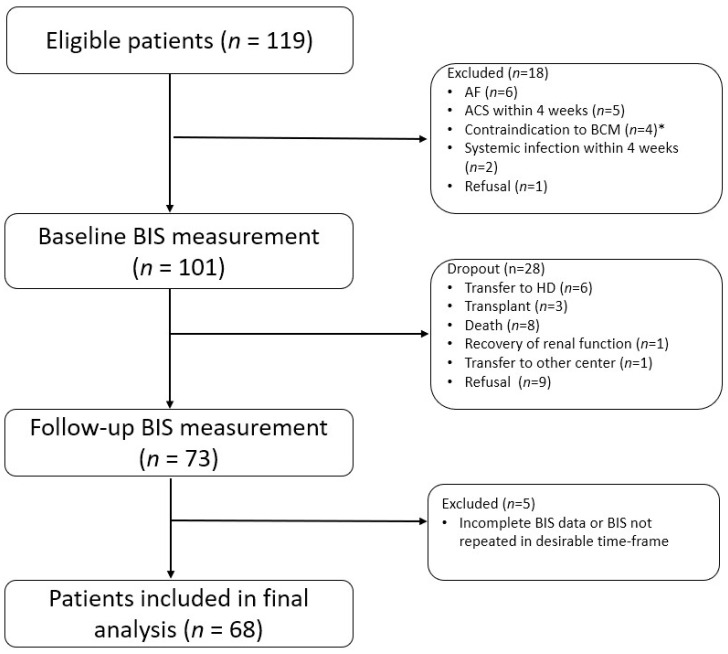
Flow diagram of the study. * Contraindications included pacemaker implantation (*n* = 1) and metallic prosthesis (*n* = 3). Abbreviations: ACS, acute coronary syndrome; AF, atrial fibrillation; BIS, bioimpedance spectroscopy; HD, hemodialysis.

One hundred and nineteen incident PD patients were screened from December 2018 to March 2021, from whom eighteen were excluded (Figure 1). Among the 101 patients who underwent baseline BIS, 54 (53.5%) were male and the mean age of the entire cohort was 59.4 ± 12.3 years. The prevalence of diabetes was 59.4%. Nineteen (18.8%) and forty-one (40.6%) patients were classified as overweight (BMI 23–24.9 kg/m^2^) and obese (BMI ≥ 25.0 kg/m^2^), respectively. Male patients had significantly higher BMI. There was no significant difference in comorbidity burden between male and female patients. Upon initiation of PD, 21 (20.8%) patients used automated peritoneal dialysis (APD). While 35 (34.7%) patients were treated with biocompatible (neutral pH) PD solution, only 2 (2.0%) used icodextrin as part of the initial dialysis regimen. Although both genders had similar small solute clearance (Kt/V), male patients had greater residual renal function. Table 1 summarized the baseline demographics, biochemical parameters and dialysis characteristics of the baseline cohort.

### 3.2. Relationship between Body Composition and Cardiac Markers

At baseline, 101 patients underwent concurrent BIS and echocardiography. Volume overload was highly prevalent in incident PD patients (mean RHI = 20.2 ± 11.1%). Twenty-one (20.8%) and seventy (69.3%) patients were considered to have mild (RHI > 7 to 15%) and severe (RHI > 15%) volume overload, respectively (Table 1). LTI had a weak correlation with NPNA (r = 0.22, *p* = 0.03) but did not correlate with serum albumin (r =0.16, *p* =0.10). There was no association between FTI and serum albumin (r = 0.01), NPNA (r = −0.05) or serum cholesterol (r = −0.01) (all *p*-values > 0.05). Male patients had comparable volume status and FTI with their female counterparts, but significantly higher LTI. In addition, the majority of patients (96.0%) had preserved LVEF (≥50%). There was no significant difference between systolic function and NT-proBNP between male and female patients.

After adjustment of age, gender, and BSA, RHI was significantly associated with E/e’ (beta = 0.41, *p* < 0.0001), LAVi (beta =0.35, *p* < 0.0001), and LVEDD (beta = 0.25, *p* = 0.02). As expected, RHI was also positively correlated with NT-proBNP (Table 2).

Univariate analysis showed that LVEDD significantly correlated with LTI (r = 0.28, *p* = 0.005). However, this association became insignificant (beta = 0.07, *p* = 0.39) when age, gender and BSA were included in multivariable regression, suggesting they were potential confounders. Moreover, neither E/e’ nor LAVi was associated with LTI (Table 2). Similarly, there was no significant association between any echocardiographic parameters and FTI (Table 2). Nonetheless, FTI was negatively associated with NT-proBNP (beta = −0.22, *p* = 0.03) (Table 2).

### 3.3. Change in Body Composition over Time

The second BIS was performed after a median of 12.9 (IQR 12.2–14.4) months. During this period, eight patients died (75% due to cardiovascular diseases). Moreover, six patients were transferred to long-term hemodialysis and three received kidney transplant. After further exclusion of 5 patients whose BIS data were incomplete or BIS was performed out of the desired timeframe, 68 patients were included in the final analysis (Figure 1). Patients that were excluded had no significant difference from those retained in the final analysis in terms of baseline demographics, body composition and echocardiographic parameters, except weekly Kt/V was lower in the former group (*p* = 0.01) (Table S1).

Among the 68 patients with repeated BIS measurements, BMI (23.8 [22.3–28.6] vs. 23.6 [21.6–26.9] kg/m^2^, *p* = 0.004) and FTI (9.4 ± 4.6 vs. 8.6 ± 4.7 kg/m^2^, *p* = 0.02) increased significantly. On the contrary, there was no significant change in LTI (14.6 ± 3.2 [follow-up] vs. 14.5 ± 2.7 [baseline] kg/m^2^, *p* = 0.78). There was also no significant change in the proportion of patients with muscle wasting (LTI < 10th percentile) from baseline to follow-up BIS (11.8% vs. 11.8%, *p* = 1.0). Despite a significant decrease in RHI from first (20.1 ± 10.9%) to second (16.7 ± 10.2%) assessment (mean difference = −3.5%, *p* = 0.02), there remained a substantial portion of patients who were in severe fluid overload (51.5%, RHI > 15%). For PD prescription, 3 patients switched from continuous ambulatory peritoneal dialysis (CAPD) to APD, and 14 patients were newly treated with icodextrin throughout the study period.

### 3.4. Predictors of Change in Lean Tissue and Fat Tissue Mass

To identify the potential predictors of change in LTI and FTI, four linear regression models were constructed using baseline parameters and serial parameters, respectively.

**Figure 2:**
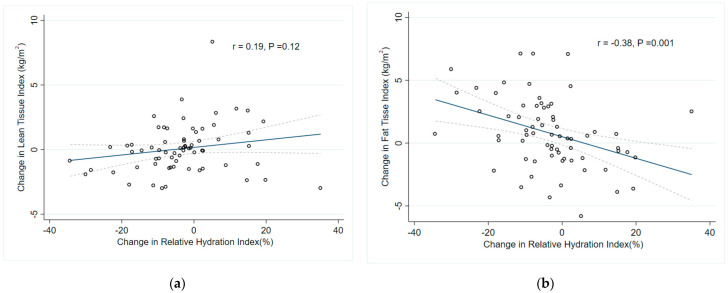
The effect of change in hydration status on (**a**) change in lean tissue (r = 0.19, *p* = 0.12); (**b**) and change in fat tissue (r = −0.38, *p* = 0.001). Dotted line represented 95% confidence interval of linear regression. FTI, fat tissue index; LTI, lean tissue index; RHI, relative hydration index.

Univariable analysis showed that lower baseline serum albumin, high baseline total cholesterol, higher RHI and lower FTI were associated with lean tissue wasting. However, multivariable regression model using baseline parameters showed that only baseline FTI (beta = 0.26, 95% confidence interval (CI) 0.04–0.48, *p* = 0.02) remained independent predictor of change in LTI (adjusted R^2^ = 0.169, *p* = 0.003) (Table 3). When we included time-averaged predictors in the multivariable regression model (Table 4), increase in mean serum albumin (beta = 0.21, 95% CI 0.06–0.37, *p* = 0.01) and decrease in FTI (beta = −0.71, 95% CI −0.87–−0.55, *p* < 0.0001) from first to second BIS were associated with a significant increase in LTI (adjusted R^2^ = 0.633, *p* < 0.0001). Neither peritoneal glucose exposure nor change in RHI was related to the change in LTI (Figure 2a).

**Figure 3:**
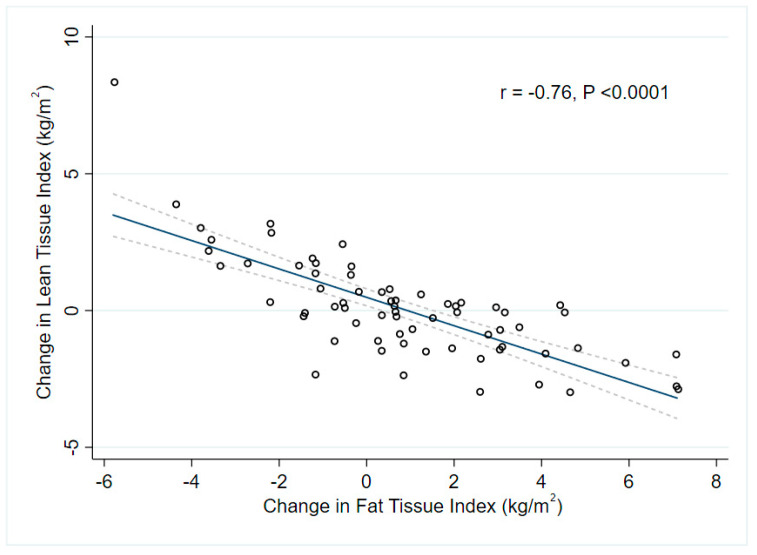
Gain in fat mass was strongly associated with loss in lean mass (r = −0.76, *p* < 0.0001). Dotted line represented 95% confidence interval of linear regression. FTI, fat tissue index; LTI, lean tissue index.

On the other hand, univariate analysis revealed a significant association between an increase in FTI and baseline peritoneal glucose exposure, baseline RHI, but an inverse association with baseline RHI. However, multivariable regression model demonstrated that lower baseline FTI (beta = −0.29, 95% CI −0.52–−0.06, *p* = 0.01) and higher baseline RHI (beta = 0.28, 95% CI 0.05–0.51, *p* = 0.02) independently predicted gain in fat mass (adjusted R^2^ = 0.165, *p* = 0.001) (Table 3). When we included time-averaged predictors in the multivariable regression model (Table 4), reduction in LTI (beta = −0.72, 95% CI −0.76–−0.67, *p* < 0.0001) and reduction in RHI (beta = −0.35, 95% CI −0.37 –−0.33, *p* < 0.0001) from first to second BIS were associated with a significant increase in FTI (adjusted R^2^ =0.633, *p* < 0.0001) (Figure 2b and Figure 3). There was no significant association between change in FTI, change in PD modality, use of icodextrin and peritoneal glucose exposure.

A total of 67 hospital admissions occurred in 30 patients (44.1%) during the study period. We conducted a sensitivity analysis by repeating linear regression to determine the change in LTI and FTI after excluding patients with a history of hospitalization as it may modify body composition. Interestingly, while the association between mean CRP and change in LTI only reached marginal significance in our primary analysis, their association became significant (beta = −0.27, *p* = 0.001) after the exclusion of patients with hospitalization (Table S2). The rest of multivariable regression analysis remained similar.

## 4. Discussion

Assessment of body composition is an essential component of nutrition assessment in dialysis patients. Dynamic body composition changes, including volume overload, gain in adiposity, and muscle wasting, may either occur alone or simultaneously after initiation of PD. Published evidence demonstrated that BIS could help nephrologists to differentiate between LTM, ATM and OH in clinical practice. In the present study, we showed that the measurement of lean tissue and adipose tissue was not affected by volume status assessed by echocardiography. Moreover, baseline demographics, biochemical parameters and PD prescriptions did not predict change in body compositions over a median of 12.9 months, Nevertheless, gain in fat mass was significantly associated with muscle wasting. Additionally, fluid accumulation independently predicted a decrease in adiposity but not lean tissue mass.

The validity of the assessment of LTM by bioimpedance technique was challenged by Konings et al. who showed that LBM strongly correlated with LVEDD in 39 PD patients [18]. However, LVEDD was not a sensitive marker of hydration status since it took time for cardiac remodeling to occur. Additionally, dilated ventricles may simply reflect pre-existing cardiac diseases instead of fluid overload. Of note was that severe ventricular dilatation was already present in 23% of patients [18]. By contrast, our cohort (*n* = 101) had excluded known valvular heart disease and only 7.9% had a history of congestive heart failure. Importantly, E/e’ and LVAi were shown to accurately reflect LV filling pressure, and their elevation indicated an increase in preload [19,20]. Our study showed that neither FTI nor LTI was associated with E/e’ and LAVi. We also verified the relationship between these echocardiographic parameters with fluid status by confirming a significant positive association with RHI (Table 2). Although univariable analysis showed a correlation between LVEDD and LTI (r = 0.28, *p* = 0.005), this was no longer present after adjustment for age, gender and BSA. Indeed, the fact that age (r = −0.30, *p* = 0.002), gender (r = 0.65, *p* < 0.0001), and BSA (r = 0.53, *p* < 0.0001) was associated with LTI suggested that they were potential confounders, as they were also closely related to LVEDD such that normal values of LVEDD should be stratified by age and gender, as well as indexed by BSA [27]. The failure to adjust for these factors may explain the discrepancy between our results and those by Konings et al., especially the fact that their cohort was predominantly male (73%) [18]. While LTI was not related to NT-proBNP, it might be unexpected to see an inverse association between FTI and NT-proBNP. However, the level of NT-proBNP may also be affected by renal clearance. A post hoc analysis that adjusted age, gender, and residual glomerular filtration rate revealed that the relationship between FTI and NT-proBNP fell short of significance (beta = −0.20, *p* = 0.06). Considering the lack of an association with other echocardiographic parameters, it was less likely that the measurement of adipose tissue would be affected by hydration status. These findings were highly relevant in clinical practice as a substantial proportion of PD patients remained in fluid overload after the first year, which was previously believed to confound assessment of LTM and ATM.

Apart from the selection of echocardiographic parameters, another important difference was the choice of bioimpedance technique. Multifrequency BIA [18] adopted a two-compartment model which was composed of fat mass and fat-free mass. This model assumed fat mass to be anhydrous and excessive fluid was contained in the fat-free mass, leading to a highly variable hydration state in this compartment. In contrast, our study used a multifrequency BIS (Body Composition Monitor), which applied a three-compartment model that assumed a fixed hydration constant in both LTM and ATM, with excessive ECW being considered separately in a compartment called ‘OH’ [15,30]. Therefore, the assessment of muscle and fat mass by BIS should have a theoretical advantage such that the measurements seemed to be less biased by hydration status. On the other hand, a recent study that compared BIS to DEXA suggested that difference (if any) in body composition measurement between these two modalities had a significant correlation with OH [31]; but this finding could not be replicated in another study with similar design [32]. To this end, it is important to recognize that the estimation of LBM by DEXA was in fact strongly influenced by hydration status [33]. In other words, a ‘gold standard’ for assessing body composition in ESRD patients may not truly exist.

A number of previous studies described the trajectory of body composition measured by bioimpedance techniques in PD patients [17,34,35,36,37]. The largest up-to-date observational study (Initiative for Patient Outcomes in Dialysis-Peritoneal Dialysis (IPOD-PD)) reported a progressive weight gain and increase in adiposity, which was accompanied by a mild decrease in lean tissue over time in 1054 incident PD patients [37]. This was compatible with our findings except that we did not observe a significant change in LTI. This may not be surprising because the most significant loss in lean tissue seemed to occur after the second year [37]. In addition, 57% of patients were in fluid overload (defined as RHI > 7%) at the beginning of dialysis, which tended to improve after the first year to 48% and remained stabilized [12]. It was believed that BIS findings at the start of PD (which may not always be available before dialysis) prompt the clinicians to intensify volume control. Thus, a similar decline in RHI was observed in our study; yet 82.4% of patients remained in fluid overload after one year, which was probably accounted for by a worse volume status at baseline (mean RHI 20.2% vs. 9.7% (IPOD-PD)) [12]. Similar studies in a pediatric population were limited. In a small cohort of children treated with APD, 48% were overhydrated (RHI > 7%) by BIS, which persisted in a 6-month follow up [38]. An earlier study using bioimpedance vector analysis showed that nutrition parameter (phase angle) improved after starting CAPD [39].

The investigators of the IPOD-PD study reported that age, dialysis vintage, and use of polyglucose or hypertonic PD solution were associated with an increase in FTI and a decrease in LTI [37], while another group found no association between change in body composition with peritoneal transporter status and dialysate glucose load in selected APD patients [17]. Since polyglucose or hypertonic solution was frequently used to treat fluid overload, their association with LTI and FTI may be confounded by volume status. However, this was not explored in IPOD-PD study [37]. Kang et al. investigated the impact of volume status (using edema index [ECW divided by total body water (TBW)] derived from BIA) on body composition of 366 incident PD patients [34]. They found that overhydration predicted the development of sarcopenia but had a neutral effect on fat mass [34]. However, their results should be interpreted with caution because muscle wasting was associated with a decrease in intracellular water. Hence, low BMI or muscle wasting can lead to overestimation in edema index even in the absence of expansion of ECW. This mathematical coupling could be circumvented by the three-compartment model of BIS. Calculation of OH by a fixed hydration ratio resulted in less bias than using ECW:TBW across subjects with variable body composition [21]. In contrast to the findings of Kang et al., our study showed that volume status (both baseline and change) did not predict change in LTI in 1 year. We postulated the relatively short period of follow up and the initial improvement in volume status could potentially attenuate the effect of volume status on muscle mass in our study. A recent longitudinal study of 269 PD patients over approximately 4 years revealed that an increase in time-averaged RHI was associated with a progressive decline in LTI and FTI by a linear mixed model [40]. It was believed that chronic inflammation in ESRD mediated muscle breakdown by activating myostatin and other catabolic pathways [41]. Our study also suggested a possible role of inflammation on muscle mass. Baseline and mean CRP had a trend towards negative correlation with LTI (Table 3 and Table 4); persistent hypoalbuminemia (beta = 0.21, *p* = 0.01), which was common in chronic inflammation, significantly predicted the decline in LTI. We excluded patients with hospitalization in the sensitivity analysis to minimize confounding by acute illness. Notably, mean CRP became a significant predictor of lean tissue loss (beta = −0.27, *p* = 0.001). Interestingly, fluid overload may aggravate systemic inflammation via bacterial translocation through edematous bowel (‘the leaky gut’) [6,42]. In addition to the volume overload and inflammation, hyperphosphatemia was also linked to cardiovascular mortality. As a result, dietary phosphate restriction and judicious use of phosphate binders were also important in the management of PD patients [43,44].

Our study showed that weight gain during early PD was mainly driven by a gain in fat mass and was less likely linked to the accumulation of fluid and increase in muscle mass. Conversely, a stable BW or BMI did not guarantee a stable body composition, as gain in fat mass was often associated with muscle wasting. Moreover, most of the baseline demographics, biochemical parameters and PD prescriptions did not predict a change in body compositions (Table 3). Lower FTI at baseline was associated with a progressive increase in adiposity but a decline in lean mass. The former could be possibly attributed to improvements in uremic symptoms at the beginning of dialysis [10]. Nevertheless, the low adjusted R^2^ (Table 3) implied that the majority of variance in the dependent variable was not explained by the present model. On the contrary, we showed that increase in adiposity significantly predicted muscle wasting (beta = −0.71, *p* < 0.0001), which was in keeping with previous studies [17,35,36], The model-fit remained respectable (adjusted R^2^ = 0.633) even after adjustment for nutrition and inflammatory status (Table 4). In fact, adipose tissue secretes an array of proinflammatory cytokines, of which the most common one is leptin. An earlier study showed the serum leptin strongly correlated with fat mass [45]. PD patients who experienced LBM loss had significantly higher initial CRP and remarkable increase in serum leptin [45]. As such, an increase in fat mass in early PD provided an abundant source of leptin, which suppressed appetite by acting on the hypothalamic axis, and contributed to muscle wasting together with inflammation. Our recent study revealed that adipocyte expression of another adipokine, namely zinc alpha−2-glycoprotein, also predicted a decrease in LTM [46].

We also showed that fluid accumulation contributed to a loss in fat mass (beta = −0.35, *p* < 0.0001). The underlying mechanism of such association may potentially be explained by the experiment of Luce et al., who reported that natriuretic peptides activated lipolysis and browning of fat by increasing the synthesis of uncoupling protein 1 in mouse model [47]. Therefore, fluid overload, which was often accompanied by elevation of natriuretic peptides and hypoalbuminemia, may play a role in pathogenesis of protein energy wasting. This may also account for the inverse correlation between NT-proBNP and FTI at baseline (Table 2).

The latest clinical practice guideline for nutrition from National Kidney Foundation’s Kidney Disease Outcomes Quality Initiative (KDOQI) cautioned about the use of bioimpedance to assess body composition in PD patients [9], partly related to potential bias from fluid status [18]. Our study showed that the measurement of muscle and adiposity were not affected by hypervolemia, thereby supporting the potential use of BIS in nutrition assessment in PD patients. On the other hand, subjective global assessment (SGA) was a well-established index that was recommended for evaluating nutrition in ESRD patients [9]. Studies showed poor agreement between SGA and BIS (defined as LTI <10th percentile) in determining malnutrition [31,48]. 23% of PD patients with normal SGA indeed in fact had LTI below the 10th percentile [31]. It should however be noted that low LTI per se remained an independent predictor of mortality after adjustment of serum albumin and comorbidities [17,35]. Importantly, longitudinal LTI loss was a stronger risk factor of death compared with low LTI at baseline [35]. Therefore, we believed BIS plays a complementary role to SGA in nutrition assessment [48]. This was especially true when clinical assessment of subcutaneous fat and muscle (the core elements of SGA) became less precise in obese patients, whose bony landmarks may be less prominent. Moreover, an abnormal SGA score did not inform clinicians on which body compartment required attention.

Our study had several limitations. First, the single-center design and small sample size limited the generalizability of the findings of our study. Nevertheless, our observations on the change in body composition in the early stage of PD were consistent with published studies [17,36,37]. Our study also added value to the existing literature by showing an inverse association between volume overload and adiposity loss. Second, we did not quantify fat mass separately into visceral and subcutaneous compartments, which appeared to carry different prognostic implications in ESRD patients [49]. Third, our study did not include SGA or physical performance (which was closely related to sarcopenia). Fourth, the drop-out rate was relatively high (32.7%) in this prospective cohort. This may be at least partially attributed to the global pandemic due to coronavirus disease (COVID−19), which rendered more refusal (*n* = 9) or follow-up BIS out of the desired timeframe (*n* = 5). Having said that, there were no significant differences in the baseline demographics and body composition parameters between included and excluded patients.

## 5. Conclusions

PD patients encounter complex changes in body composition which may not be adequately reflected by traditional anthropometric parameters. Based on a physiological three-compartment model, BIS allows concomitant assessment of volume and nutrition status that provide important prognostic information to clinicians. Our study showed that this approach was feasible by demonstrating that LTI and FTI were not biased by volume status gauged by echocardiography. Repeated BIS measurements showed that progressive fluid accumulation may contribute to loss in fat mass and hypoalbuminemia, both of which were the hallmark of protein energy wasting. Therefore, BIS should be considered as part of the comprehensive nutrition assessment of PD patients, alongside with SGA, muscle strength and physical performance [30]. Notwithstanding the controversial outcomes of BIS-guided fluid management [50], the role of BIS-guided nutritional intervention remains uncertain and deserves further study.

## Figures and Tables

**Table 1:** Baseline demographics, dialysis characteristics, body composition and echocardiographic parameters of incident peritoneal dialysis patients.

	Patients with Baseline BIS (*n* = 101)	Male (*n* = 54)	Female (*n* = 47)	*p*-Value *
Age (year)	59.4 ± 12.3	57.8 ± 12.7	61.2 ± 11.7	0.16
Systolic blood pressure (mmHg)	148.6 ± 18.4	146.8 ± 18.9	150.7 ± 17.7	0.29
Diastolic blood pressure (mmHg)	79.6 ± 11.3	80.1 ± 11.3	79.0 ± 11.5	0.66
BW (kg)	61.8 (53.9 to 75.6)	67.3 (61.8 to 83.2)	55.2 (50.9 to 58.9)	<0.0001
BMI (kg/m^2^)	23.9 (21.6 to 28.1)	25.0 (22.4 to 30.2)	22.7 (21.0 to 25.8)	0.02
Causes of renal failure, no. of cases (%)				0.21
Diabetic nephropathy	55 (54.5%)	33 (61.1%)	22 (46.8%)	
Glomerulonephritis	21 (20.8%)	9 (16.7%)	12 (25.5%)	
Hypertensive nephrosclerosis	11 (10.9%)	5 (9.3%)	6 (12.8%)	
Polycystic kidney	5 (5.0%)	1 (1.9%)	4 (8.5%)	
Comorbidities, no. of cases (%)				
Diabetes	60 (59.4%)	35 (64.8%)	25 (53.2%)	0.24
Ischemic heart disease	11 (10.9%)	6 (11.1%)	5 (10.6%)	0.94
Congestive heart failure	8 (7.9%)	5 (9.3%)	3 (6.4%)	0.72
Cerebrovascular disease	7 (6.9%)	6 (11.1%)	1 (2.1%)	0.12
Charlson’s Comorbidity Index	5.2 ± 1.9	5.2 ± 1.8	5.1 ± 2.1	0.81
Laboratory parameters				
Hemoglobin (g/dL)	9.8 ± 1.4	10.0 ± 1.4	9.5 ± 1.5	0.10
Albumin (g/L)	31.7 ± 4.5	32.4 ± 4.3	30.8 ± 4.6	0.07
Creatinine (µmol/L)	792 (640 to 936)	766 (615 to 950)	824 (654 to 936)	0.43
Fasting glucose	5.9 ± 3.0	5.7 ± 1.6	6.2 ± 4.0	0.42
Total cholesterol (mmol/L)	4.2 ± 1.2	3.7 ± 1.0	4.8 ± 1.2	<0.0001
C-reactive protein (mg/L)	2.0 (0.7 to 5.8)	1.7 (0.7 to 5.8)	2.2 (0.8 to 6.0)	0.80
NT-proBNP (pg/mL)	409.8 (184.8 to 857.4)	382.3 (161.8 to 765.4)	423.5 (206.7 to 1031.8)	0.29
Dialysis Characteristics				
Peritoneal glucose exposure (g/day)	93.5 ± 21.6	92.9 ± 21.9	94.2 ± 21.4	0.75
APD, no. of cases (%)	22 (21.8%)	13 (24.1%)	9 (19.2%)	0.55
D/P creatinine at 4 h	0.65 ± 0.12	0.64 ± 0.13	0.66 ± 0.11	0.34
Dialysis adequacy				
Weekly total Kt/V	2.04 (1.63 to 2.40)	2.00 (1.65 to 2.23)	2.04 (1.56 to 2.54)	0.47
Residual GFR (ml/min/1.73 m^2^)	3.74 (1.71 to 6.55)	5.68 (3.40 to 8.41)	2.30 (1.48 to 3.84)	<0.0001
Residual urine volume (L/day)	1.15 ± 0.73	1.36 ± 0.78	0.92 ± 0.58	0.004
NPNA (g/kg/day)	1.05 ± 0.27	1.06 ± 0.25	1.04 ± 0.30	0.68
Echocardiographic measurements				
EF (%)	59.5 ± 6.7	58.8 ± 7.4	60.4 ± 5.8	0.26
E/e’	13.8 (10.9 to 18.0)	13.1 (9.9 to 17.3)	15.4 (11.2 to 20.4)	0.07
LAVi (ml/m^2^)	34.8 (27.9 to 43.4)	34.9 (28.3 to 39.7)	34.6 (27.9 to 43.7)	0.92
LVEDD (mm)	46.4 ± 6.5	47.6 ± 6.9	44.9 ± 5.7	0.03
Body composition				
OH (liter)	3.3 (1.9 to 5.4)	3.6 (2.4 to 5.5)	3.2 (1.8 to 4.1)	0.14
RHI (%)	20.2 ± 11.1	19.4 ± 10.8	21.1 ± 11.6	0.46
LTI (kg/m^2^)	14.3 ± 2.8	16.0 ± 2.4	12.4 ± 1.8	<0.001
FTI (kg/m^2^)	9.1 ± 4.8	8.5 ± 4.6	9.7 ± 5.0	0.20

* Comparison between male and female. Abbreviations: APD, automated peritoneal dialysis; BIS, bioimpedance spectroscopy; BW, body weight; BMI, body mass index; D/P creatinine, ratio of dialysate to plasma concentration of creatinine; EF, ejection fraction; E/e’, early trans-mitral velocity to tissue Doppler mitral annular early diastolic velocity ratio; FTI, fat tissue index; GFR, glomerular filtration rate; Kt/V. total urea clearance (peritoneal and urine); LAVi, left atrial volume index; LTI, lean tissue index; LVEDD, left ventricular end diastolic volume; NPNA, normalized protein nitrogen appearance; NT-proBNP, *N*-terminal pro-brain natriuretic peptide; OH, volume of overhydration; RHI, relative hydration index.

**Table 2:** Relationship between body composition parameters and cardiac markers (NTpro-BNP and echocardiographic parameters) (*n* = 101).

	Baseline Lean Tissue Index (kg/m^2^)		Baseline Fat Tissue Index (kg/m^2^)		Baseline Relative Hydration Index (%)	
	β	*p*-Value	β	*p*-Value	β	*p*-Value
E/e’	0.12 ^a^	0.11	0.05 ^a^	0.29	0.41 ^a^	<0.0001
LAVi	0.07 ^a^	0.38	−0.06 ^a^	0.38	0.35 ^a^	<0.0001
LVEDD	0.07 ^a^	0.39	−0.03 ^a^	0.72	0.25 ^a^	0.02
EF (%)	0.01 ^a^	0.90	−0.03 ^a^	0.66	−0.10 ^a^	0.32
ln(NT-proBNP)	0.01 ^b^	0.90	−0.22 ^b^	0.03	0.52 ^b^	<0.0001

^a^ Adjusted for age, sex, and body surface area; ^b^ Adjusted for age and sex. Abbreviations: EF, ejection fraction; E/e’, early trans-mitral velocity to tissue Doppler mitral annular early diastolic velocity ratio; LAVi, left atrial volume index; LVEDD, left ventricular end-diastolic diameter; NTproBNP, *N*-terminal pro-brain natriuretic peptide.

**Table 3:** Univariable and multivariable regress analysis to predict change in lean tissue index (LTI) and fat tissue index (FTI) from baseline parameters (*n* = 68).

	Change in LTI (kg/m^2^)				Change in FTI (kg/m^2^)			
	Univariable Analysis		Multivariable Analysis ^a^		Univariable Analysis		Multivariable Analysis ^b^	
	β	*p*-Value	β (95%CI)	*p*-Value	β	*p*-Value	β (95% CI)	*p*-Value
Demographics								
Age	0.04	0.76	-	-	−0.07	0.60	^-^	-
Male gender	0.08	0.52	-	-	−0.05	0.69	^-^	-
CCI	−0.14	0.27	-	-	0.12	0.35	^-^	-
Laboratory parameters								
Albumin	0.31	0.01	0.20 (−0.05–0.44)	0.12	−0.21	0.08	^-^	-
ln(CRP)	−0.24	0.046	−0.20 (−0.44–0.03)	0.09	0.14	0.24	^-^	-
FBG	−0.02	0.88	-	-	0.03	0.83	^-^	-
Total cholesterol	−0.27	0.03	−0.19 (−0.43–0.04)	0.11	0.12	0.33	^-^	-
Dialysis factors								
APD (vs. CAPD)	−0.02	0.91	-	-	0.04	0.74	-	-
Peritoneal glucose exposure	−0.22	0.07	-	-	0.27	0.02	-	-
D/P_Cr_	−0.12	0.33	-	-	0.10	0.45	-	-
Weekly total Kt/V	−0.09	0.49	-	-	0.14	0.26	-	-
Residual urine volume	0.10	0.41	-	-	−0.16	0.19	-	-
Body composition								
Baseline LTI	−0.11	0.39	-	-	0.06	0.63	-	-
Baseline FTI	0.22	0.07	0.26 (0.04–0.48)	0.02	−0.34	0.005	−0.29 (−0.52–−0.06)	0.01
Baseline RHI	−0.30	0.01	-	-	0.33	0.01	0.28 (0.05–0.51)	0.02
Baseline BMI	0.08	0.51	-	-	−0.22	0.07	-	-

^a^ Adjusted for age, sex, serum albumin, ln(CRP), total cholesterol, peritoneal glucose exposure, baseline FTI and RHI (adjusted R^2^= 0.169); ^b^ Adjusted for age, sex, serum albumin, peritoneal glucose exposure, baseline FTI, RHI and BMI (adjusted R^2^ = 0.165). Abbreviations: APD, automated peritoneal dialysis, APD; BMI, body mass index; CAPD, continuous ambulatory peritoneal dialysis; CCI, Charlson comorbidity index; CI, confidence interval; CRP, C-reactive protein; D/P _Cr,_ ratio of dialysate to plasma concentration of creatinine after standard peritoneal equilibration test; FBG, fasting blood glucose; FTI, fat tissue index; RHI, relative hydration index.

**Table 4:** Univariable and multivariable regress analysis to predict change in lean tissue index (LTI) and fat tissue index (FTI) from serial parameters (*n* = 68).

	Change in LTI (kg/m^2^)				Change in FTI (kg/m^2^)			
	Univariable Analysis		Multivariable Analysis ^a^		Univariable Analysis		Multivariable Analysis ^b^	
	β	*p*-Value	β (95%CI)	*p*-Value	β	*p*-Value	β (95% CI)	*p*-Value
Demographics								
Age	0.04	0.76	-	-	−0.07	0.60	^-^	-
Male gender	0.08	0.52	-	-	−0.05	0.69	^-^	-
Baseline CCI	−0.14	0.27	-	-	0.12	0.35	^-^	-
Laboratory parameters								
Mean albumin	0.33	0.01	0.21 (0.06–0.37)	0.01	−0.15	0.22	^-^	-
Mean ln(CRP)	−0.29	0.02	−0.12 (−0.28–0.03)	0.10	0.20	0.11	^-^	-
Mean FBG	0.09	0.47	-	-	−0.04	0.74	^-^	-
Mean total cholesterol	−0.23	0.06	-	-	0.15	0.22	^-^	-
Dialysis factors								
New APD user	−0.03	0.78	-	-	0.11	0.39	-	-
New icodextrin user	−0.16	0.19	-	-	0.06	0.64	-	-
Time-averaged peritoneal glucose exposure	−0.08	0.54	-	-	0.10	0.40	-	-
Time-averaged weekly total Kt/V	−0.10	0.47	-	-	0.12	0.37	-	-
Time-averaged residual urine volume	0.07	0.60	-	-	−0.03	0.83	-	-
Body composition								
Change in LTI	-	-	-	-	−0.76	<0.0001	−0.72 (−0.76–−0.67)	<0.0001
Change in FTI	−0.76	<0.0001	−0.71 (−0.87–−0.55)	<0.0001	-	-	-	-
Change in RHI	0.19	0.12	-	-	−0.38	0.001	−0.35 (−0.37–−0.33)	<0.0001
Change in BMI	0.08	0.50	-	-	0.496	<0.0001	0.59 (0.54–0.64)	<0.0001

^a^ Adjusted for age, sex, mean serum albumin, mean ln(CRP), mean total cholesterol, change in FTI (adjusted R^2^ = 0.633); ^b^ Adjusted for age, sex, change in LTI, change in RHI, and change in BMI (adjusted R^2^ = 0.967). Abbreviations: APD, automated peritoneal dialysis, APD; BMI, body mass index; CAPD, continuous ambulatory peritoneal dialysis; CCI, Charlson comorbidity index; CI, confidence interval; CRP, C-reactive protein; FBG, fasting blood glucose; FTI, fat tissue index; RHI, relative hydration index.

## Data Availability

The data underlying this article will be shared on reasonable request to the corresponding author.

## Supplementary Materials

The following supporting information can be downloaded at: https://www.mdpi.com/article/10.3390/nu14194076/s1: Table S1. Baseline demographics, dialysis characteristics, body composition and echocardiographic parameters of incident peritoneal dialysis patients; Table S2. Univariable and multivariable regress analysis to predict change in LTI and FTI from serial parameters (*n* = 38).
