# Supplementary material for: The Impact of Volume Overload on the Longitudinal Change of Adipose and Lean Tissue Mass in Incident Chinese Peritoneal Dialysis Patients
Source: Nutrients. 2022 Sep 30;14(19):4076. doi: 10.3390/nu14194076 (PMC9571726; doi:10.3390/nu14194076)
Supplement: Supplementary file 1 [file nutrients-14-04076-s001.zip › nutrients-1931838-supplementary.pdf]

Table S1. Baseline demographics, dialysis characteristics, body composition and echocardiographic parameters of incident peritoneal dialysis patients

	Patients with baseline BIS (n=101)	Patients with baseline and follow-up BIS (n=68)	Patients excluded after baseline BIS (n=33)	P-value*
Male (%)	54 (53.5%)	35 (51.5%)	19 (57.6%)	0.56
Age (year)	59.4 ± 12.3	60.6 ± 11.5	56.8 ± 13.6	0.18
Systolic blood pressure (mmHg)	148.6 ± 18.4	147.6 ± 18 0	150.5 ± 19.4	0.46
Diastolic blood pressure (mmHg)	79.6 ± 11.3	79.1 ± 11.2	80.7 ± 11.8	0.51
BW (kg)	61.8 (53.9 to 75.6)	59.6 (54.0 to 70.1)	66.5 (53.9 to 79.5)	0.31
BMI (kg/m^2)	23.9 (21.6 to 28.1)	23.6 (21.6 to 26.9)	25.4 (21.8 to 30.6)	0.23
Causes of renal failure, no. of cases (%)				0.44
Diabetic nephropathy	55 (54.5%)	36 (52.9%)	19 (57.6%)	
Glomerulonephritis	21 (20.8%)	13 (19.1%)	8 (24.2%)	
Hypertensive nephrosclerosis	11 (10.9%)	9 (13.2%)	2 (6.1%)	
Polycystic kidney	5 (5.0%)	3 (4.4%)	2 (6.1%)	
Comorbidities, no. of cases (%)				
Diabetes	60 (59.4%)	40 (58.8%)	20 (60.6%)	0.86
Ischemic heart disease	11 (10.9%)	7 (10.3%)	4 (12.1%)	0.75
Congestive heart failure	8 (7.9%)	5 (7.4%)	3 (9.1%)	0.71
Cerebrovascular disease	7 (6.9%)	5 (7.4%)	2 (6.1%)	1.00
Charlson's Comorbidity Index	5.2 ± 1.9	5.2 ± 2.0	5.2 ± 1.9	0.87
Laboratory parameters				
Hemoglobin (g/dL)	9.8 ± 1.4	9.7 ± 1.4	10.0 ± 1.5	0.41
Albumin (g/L)	31.7 ± 4.5	32.1 ± 3.7	30.7 ± 4.7	0.21
Creatinine (μmol/L)	792 (640 to 936)	812 (674 to 935)	875 (604 to 944)	0.70
Fasting glucose	5.9 ± 3.0	5.8 ± 1.6	6.1 ± 4.7	0.75

Total cholesterol (mmol/L)	4.2 ± 1.2	4.3 ± 1.3	4.1 ± 1.1	0.46
C-reactive protein (mg/L)	2.0 (0.7 to 5.8)	1.6 (0.7 to 5.1)	2.6 (1.0 to 11.8)	0.11
NT-proBNP (pg/ml)	409.8 (184.8 to 857.4)	370.6 (181.4 to 836.0)	482.1 (192.8 to 1016.1)	0.35
Dialysis Characteristics				
Peritoneal glucose exposure (g/day)	93.5 ± 21.6	94.0 ± 20.4	92.5 ± 24.2	0.77
APD, no. of cases (%)	22 (21.8%)	15 (22.1%)	7 (21.2%)	0.92
D/P creatinine at 4 hour	0.65 ± 0.12	0.65 ± 0.12	0.66 ± 0.11	0.61
Dialysis adequacy				
Weekly total Kt/V	2.04 (1.63 to 2.40)	2.08 (1.72 to 2.52)	1.80 (1.44 to 2.20)	0.01
Residual GFR (ml/min/1.73m^2)	3.74 (1.71 to 6.55)	3.70 (1.77 to 7.59)	3.51 (1.36 to 5.84)	0.29
Residual urine volume (L/day)	1.15 ± 0.73	1.18 ± 0.74	1.10 ± 0.71	0.65
NPNA (g/kg/day)	1.05 ± 0.27	1.06 ± 0.26	1.02 ± 0.30	0.49
Echocardiographic measurements				
EF (%)	59.5 ± 6.7	59.9 ± 6.5	58.7 ± 7.2	0.40
E/e'	13.8 (10.9 to 18.0)	13.5 (10.9 to 17.3)	15.7 (10.5 to 19.6)	0.63
LAVi (ml/m^2)	34.8 (27.9 to 43.4)	34.8 (28.3 to 42.6)	34.6 (27.0 to 45.2)	0.87
LVEDD (mm)	46.4 ± 6.5	46.1 ± 6.0	46.9 ± 7.6	0.59
Body composition				
OH (liter)	3.3 (1.9 to 5.4)	3.2 (1.8 to 5.4)	3.8 (2.4 to 5.5)	0.41
RHI (%)	20.2 ± 11.1	20.1 ± 10.9	20.4 ± 11.7	0.91
LTI (kg/m^2)	14.3 ± 2.8	14.5 ± 2.7	13.9 ± 3.0	0.33
FTI (kg/m^2)	9.1 ± 4.8	8.6 ± 4.7	10.0 ± 5.0	0.17

*Comparison between patients with repeated BIS to patients that were excluded

Abbreviations: APD, automated peritoneal dialysis; BIS, bioimpedance spectroscopy; BW, body weight; BMI, body mass index; D/P creatinine, ratio of dialysate to plasma concentration of creatinine; EF, ejection fraction; E/e', early trans-mitral velocity to tissue Doppler mitral annular early diastolic velocity ratio; FTI, fat tissue index; LAVi, left atrial volume index; LTI, lean tissue index; NPNA, normalized protein nitrogen appearance; NT-proBNP, N-terminal pro-brain natriuretic peptide; OH, volume of overhydration; RHI, relative hydration index

Table S2. Univariable and multivariable regress analysis to predict change in LTI and FTI from serial parameters (n=38)

	Change in LTI (kg/m^2)				Change in FTI (kg/m^2)			
	Univariable analysis		Multivariable analysis		Univariable analysis		Multivariable analysis	
	β	P-value	β (95%CI)	P-value	β	P-value	β (95% CI)	P-value
Demographics								
Age	0.18	0.27	-	-	-0.18	0.28	-	-
Male gender	0.24	0.15	-	-	-0.18	0.29	-	-
CCI	-0.003	0.99	-	-	-0.001	0.99	-	-
Laboratory parameters								
Mean albumin	0.23	0.17	-	-	-0.09	0.58	-	-
Mean ln(CRP)	-0.37	0.03	-0.27 (-0.43- -0.11)	0.001	0.10	0.56	-	-
Mean FBG	0.07	0.65	-	-	-0.003	0.99	-	-
Mean total cholesterol	-0.36	0.03	-0.18 (-0.43- -0.01)	0.04	0.23	0.18	-	-
Dialysis factors								
New APD user	-0.06	0.74	-	-	0.12	0.46	-	-
New icodextrin user	-0.26	0.11	-	-	0.23	0.17	-	-
Time-averaged peritoneal glucose exposure	0.01	0.97	-	-	-0.03	0.85	-	-
Time-averaged weekly total Kt/V	-0.15	0.38	-	-	0.21	0.19	-	-

Time-averaged residual urine volume	0.19	0.27	-	-	-0.06	0.72	-	-
Body composition								
Change in LTI	-	-	-	-	-0.83	<0.0001	-0.65 (-0.72- -0.58)	<0.0001
Change in FTI	-0.83	<0.0001	-0.75 (-0.92- -0.58)	<0.0001	-	-	-	-
Change in RHI	0.51	0.001	-	-	-0.55	<0.0001	-0.29 (-0.38- -0.20)	<0.0001
Change in BMI	-0.07	0.68	-	-	0.53	0.001	0.52 (0.46-0.58)	<0.0001

a. Adjusted for age, sex, mean serum albumin, mean ln(CRP), mean total cholesterol, change in RHI, change in FTI (adjusted R^2= 0.781)
b. Adjusted for age, sex, change in LTI, change in RHI, and change in BMI (adjusted R^2= 0.969)

Abbreviations: APD, automated peritoneal dialysis, APD; BMI, body mass index; CAPD, continuous ambulatory peritoneal dialysis; CCI, Charlson comorbidity index; CI, confidence interval; CRP, C-reactive protein; FBG, fasting blood glucose; FTI, fat tissue index; RHI, relative hydration index
